# Sulfated Polysaccharides as a Fighter with Protein Non-Physiological Aggregation: The Role of Polysaccharide Flexibility and Charge Density

**DOI:** 10.3390/ijms242216223

**Published:** 2023-11-12

**Authors:** Olga N. Makshakova, Liliya R. Bogdanova, Dzhigangir A. Faizullin, Elena A. Ermakova, Yuriy F. Zuev

**Affiliations:** Kazan Institute of Biochemistry and Biophysics, FRC Kazan Scientific Center of RAS, 2/31 Lobachevsky Street, 420111 Kazan, Russia

**Keywords:** hen egg white lysozyme, amyloid fibrils, sulfated polysaccharides, chaperons, disaggregation effect, protein renaturation

## Abstract

Proteins can lose native functionality due to non-physiological aggregation. In this work, we have shown the power of sulfated polysaccharides as a natural assistant to restore damaged protein structures. Protein aggregates enriched by cross-β structures are a characteristic of amyloid fibrils related to different health disorders. Our recent studies demonstrated that model fibrils of hen egg white lysozyme (HEWL) can be disaggregated and renatured by some negatively charged polysaccharides. In the current work, using the same model protein system and FTIR spectroscopy, we studied the role of conformation and charge distribution along the polysaccharide chain in the protein secondary structure conversion. The effects of three carrageenans (κ, ι, and λ) possessing from one to three sulfate groups per disaccharide unit were shown to be different. κ-Carrageenan was able to fully eliminate cross-β structures and complete the renaturation process. ι-Carrageenan only initiated the formation of native-like β-structures in HEWL, retaining most of the cross-β structures. In contrast, λ-carrageenan even increased the content of amyloid cross-β structures. Furthermore, κ-carrageenan in rigid helical conformation loses its capability to restore protein native structures, largely increasing the amount of amyloid cross-β structures. Our findings create a platform for the design of novel natural chaperons to counteract protein unfolding.

## 1. Introduction

Protein aggregation is associated with different human diseases and represents an essential problem in biotechnology and biomedicine [1,2]. One of the aggregation forms of proteins, both intrinsically disordered or misfolded globular ones, is amyloid fibrils [2,3,4], which are coupled with neurodegenerative diseases and other malignances [5,6,7]. Developing ways to combat such aggregation is an emergent task of protein science [8,9], and by now, there is a broad collection of methods to disrupt and reverse the protein aggregation process. The prevailing cause of protein aggregation is conformational changes in misfolded proteins with stable β-sheet domains [10,11]. That is why many known procedures for inhibiting protein aggregation rely on the blocking of β-sheet contacts and promoting helix formation. For this purpose, various chemical aids are used, such as small molecule-based colloidal nanoparticles [12], steroid−quinoline hybrids [13], low molecular weight nucleoside gelators [14] and native polysaccharides [15]. Understanding the factors that control the integrity of protein aggregates requires systematic study of relevant model systems.

Recently, we have demonstrated the ability of charged polysaccharides to regulate protein post-aggregation refolding at the level of secondary, tertiary and quaternary structures [16]. We demonstrated that the addition of κ-carrageenan to gelatin increases the content of its helical structure [17]. For tetrameric glyceraldehyde-3-phosphate dehydrogenase (GAPDH), we found that κ-carrageenan can stabilize its dissociated dimeric forms [18]. In hen egg white lysozyme (HEWL), κ-carrageenan interacts with the protein β-domain and stabilizes short-living β-structures [19]. The possibility of the structural rearrangement of proteins during complex formation with polysaccharides opens the way for novel biotechnological and pharmaceutical applications [16] and new bio-inspired nanomaterials [20,21]. Furthermore, polysaccharide-based materials are promising candidates for the disaggregation of amyloid fibrils [15,22,23,24].

Amyloid fibrils isolated from living organisms are often found associated with glycosaminoglycans (GAGs) [25]—the sulfated polysaccharides of the mammalian extracellular matrix. In particular, they have been found in hepatic amyloid fibrils [26] and in Lewy bodies, a hallmark of some neurological diseases [27]. This implies the role of matrix polysaccharides as a platform for misfolded proteins’ assembly [23]. The GAG sulfation pattern is an important part of the “glycocode” that can undergo in situ remodeling, regulating the balance between healthy processes and diseases [28]. The exact role of the polysaccharide sulfation pattern in the regulation of amyloid structures is still to be unveiled. Some species of sulfated GAGs were shown to promote the formation of fibrillar structures by amyloid peptides in vitro [29,30,31]. Apparently, both the charge distribution and the carbohydrate backbone structure affect the fibril formation [32]. However, Takase at al. emphasized the importance of GAGs’ sulfation degree rather than their underlying structure in the fibrillation process [30]. Interestingly, sulfated polymers are also capable of facilitating fibril formation, but the resultant structure is rather amorphous [32]. The increasing sulfation and/or the decreasing degradation of heparan sulfate proteoglycans (HSPGs) and HS GAGs that occur in vivo due to the brain aging may lead to the formation of plaques and tangles in the AD brain, since the knockout of HS genes markedly reduces the accumulation of Aβ-fibrils in the brain. Furthermore, 6-O group sulfation and a specified length of GAG as a part of HSPGs are critical for the induction of amyloid formation [33]. On the other hand, some sulfated polysaccharides, the GAG-analogues, showed an inhibitory effect on amyloid fibril formation [24,34]. Furthermore, our recent report revealed that κ-carrageenan was able to disaggregate model amyloid fibrils and to restore the native secondary structure in a globular protein [22].

**Figure 1:**
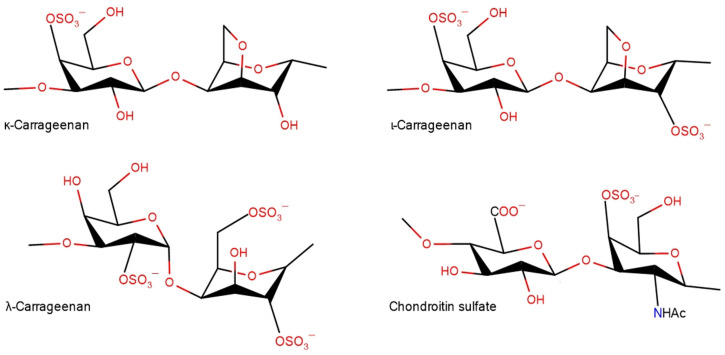
Structural scheme of sulfated polysaccharides used.

In the current work, we sought to elucidate the role of the polysaccharide sulfation pattern and chain flexibility in the propensity of sulfated polysaccharides to shift the balance between amyloid cross-β structures and native secondary structures using model HEWL fibrils. For systematical variation of the sulfated pattern, a set of carrageenans was selected (Figure 1) having one, two and three sulfated groups per disaccharide unit. The flexibility of the polysaccharide was varied by choosing its coil or helical conformation.

It is well known that in certain conditions, ι- and κ-carrageenans undergo coil-to-helix conformational transitions. In contrast, λ-carrageenan remains in the coiled state [35]. Both κ-and ι-carrageenans have a ^1^C_4_ conformation due to the 3,6-anhydro bridge, and the galactopyranosyl unit in λ-carrageenan has a ^4^C_1_ conformation. The latter is the cause of the kink, which prevents the formation of a helical structure in λ-carrageenan [36]. ι-Carrageenan revealed a coil–helix transition at high ionic strength even at low concentrations of polysaccharide [35]. An ordered dimeric three-fold double helix with 1.3 nm diameter was initially proposed for ι-carrageenan based on the X-ray diffraction pattern [37]. For κ-carrageenan, the simulations also suggest a double helix with a pitch of ~25 Å [38]. The formation of an ordered helical conformation is accompanied by an increase in the rigidity of the carrageenan chain [36]. This diversity of carrageenans’ secondary structures should influence their interactions with proteins.

HEWL was used as a classical protein model in amyloid research [39]. HEWL is structurally similar to human lysozyme (87% sequence similarity and same folding motif), which can be deposited as amyloid fibrils in kidneys [2].

The distinctive characteristic of amyloid fibrils at the level of the protein secondary structure, as shown by X-ray crystallography, is the presence of cross-β structures [40,41]. FTIR spectroscopy is a useful tool to distinguish the types of the cross-β structures of amyloids, as well as the intra- and intermolecular β-structures of other protein aggregates [42,43,44]. It was established that β-sheet IR absorbance with a maximum intensity around 1628 cm^−1^ is consistent with a cross-β morphology [36,45], while the largest and most rigid amyloids absorb near 1620 cm^−1^.

Previously, we have reported our attempts to disclose the atomistic organization in HEWL fibrils on the basis of accelerated MD simulations [46]. In the current work, we used FTIR spectroscopy to follow the perturbations in the structural organization of HEWL fibrils affected by carrageenans with different levels of sulfation. The correlation between the native-like secondary structure content and the disaggregation of HEWL fibrils at the meso-scale level was established in our previous publications using FTIR and AFM techniques, as well as CD spectroscopy [22]. The AFM images showed that long, unbranched fibrils of submicron length appeared for both the sediment and water-soluble fractions. Compared to the sizes of HEWL fibrils reported in the literature, the height of the fibrils in the sediment reached 6 nm, matching that of mature fibrils, while in the water-soluble fraction, their height was no more than ~4 nm, which is characteristic of protofibrils. The details of the secondary structure deduced from FTIR spectra revealed a relative increase in disordered elements in the water-soluble fraction, which did not affect the quality of amyloid parallel β-structures. Despite the increased fraction of disordered structures, the AFM shows that the integrity of the fibrils is not compromised. Water-soluble protofibrils were shown to be responsive for disaggregative polysaccharide action. In contrast, sediments were resistant to this action due to a tight lateral protofibril packing. In the current research, we use the water-soluble HEWL protofibrils to study the effect of complexation with polysaccharides of varying sulfation density and chain flexibility. Since the complexation of HEWL fibrils with anionic polysaccharides resulted in the formation of macroscopic gels, irrespectively to the stock solutions’ concentrations, the AFM usability for morphologic studies of these complexes at a monomolecular level was limited and, therefore, its usage was discarded. In contrast, FTIR spectroscopy has no limits in studying solid aggregates. 

The results shed light on protein–polysaccharide interactions and provide a basis for the development of approaches that regulate protein folding, which can be used as a chaperone-like procedure in medicine and pharmacy and, more broadly, for the creation of functional, inexpensive and environmentally friendly polysaccharides from renewable sources for sustainable use in the biopolymer industry [15,47,48].

## 2. Results and Discussion

### 2.1. Band Assignments

The analysis of the conformational perturbation of biopolymers as a result of protein and polysaccharide mixing was performed using FTIR spectroscopy. Infrared spectra in the Amide I region (1600–1700 cm^−1^) can be used to gain more insight into the conformation of proteins in fibrils. The Amide I band results mostly from the stretching vibrations of the peptide C=O and consists of a set of overlapping components from different protein secondary structure elements (Figure S1) [49,50]. The component at 1655 cm^−1^ is due to the absorbance of protein α-helices, and the band at 1640 cm^−1^ corresponds to the intramolecular β-structure [49]. The high frequency bands at 1674–1696 cm^−1^ are due to various types of β-turns. FTIR spectroscopy can distinguish between the intermolecular types of β-structure: namely, the parallel β-sheets consistent with the cross-β structure morphology of amyloid fibrils [36,45] and antiparallel β-structures of other types of protein aggregates. The latter has an intense band at 1615 cm^−1^ with a satellite band at 1695 cm^−1^. The former is characterized by a narrow peak in the 1620–1630 cm^−1^ region. 

**Figure 2:**
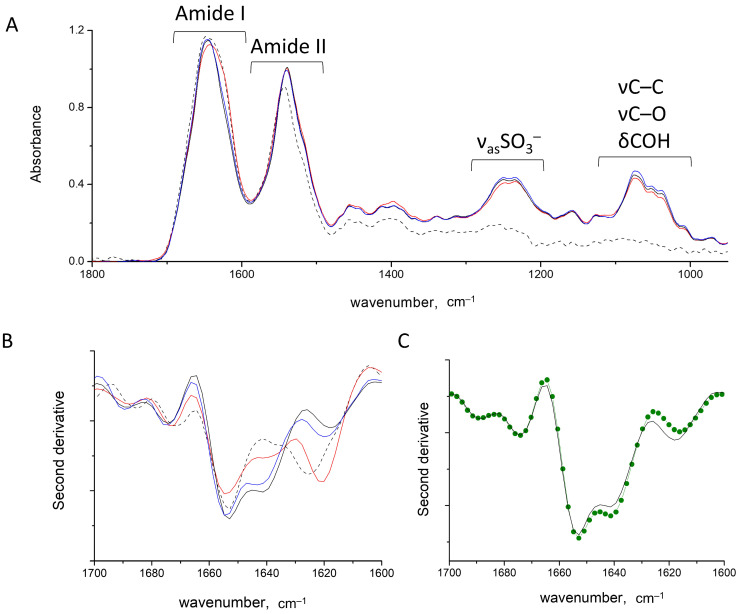
Absorbance (**A**) and second derivative (**B**) spectra of HEWL fibrils (dashed line) and their gel-like complexes with κ-carrageenan at polysaccharide/protein ratios of 0.1 (red), 0.3 (black) and 0.6 (blue). (**C**) Spectra of gel-like complexes of HEWL fibrils with κ-carrageenan (black) and native HEWL/κ-carrageenan complexes at a polysaccharide/protein ratio of 0.3 (dark green line and dots).

The FTIR spectrum of the water-soluble fraction of HEWL fibrils, proved at the meso-scale by AFM as protofibrils [22], revealed the parallel cross-β structure with absorbance at 1625 cm^−1^ (Figure 2). The parallel and antiparallel β-sheets differ primarily by the presence of a high-frequency component at 1695 cm^−1^, which is absent in the former fold. Another prominent peak located at 1655 cm^−1^ was attributed to α-helices. Furthermore, we previously demonstrated that the intensity at 1655 cm^−1^ raises at the expense of intensity at 1625 cm^−1^ in the course of salt washing out, which was explained by the detaching of the charged polypeptide termini from the protein fibrillar core [22].

In carrageenans’ spectra, the conformationally sensitive bands occupy the range of 1200–1000 cm^−1^, which consists of a number of components arising from the collective νC–C, νC–OH and δCOH vibrations of the pyranose ring [51,52,53]. The assignment of individual components is not possible; nonetheless, the spectral contour shape in the range of 1000–1100 cm^−1^ was shown to be sensitive to polysaccharide chain conformation [54,55]. In the spectrum of the κ-carrageenan helix, sharp peaks stand out at 1041 and 1068 cm^−1^ [54]. Upon the helix-to-coil transition, the bands became broader, less intensive and shifted from their initial positions (Figure S2).

### 2.2. HEWL Fibrils–Flexible κ-Carrageenan Mixtures

As follows from the spectra in Figure S2A,B, κ-carrageenan presents in coil conformation in both the initial solution and in complexes with HEWL. Coiled carrageenans are more flexible compared to helical ones [36,38]. When mixing the HEWL fibrils with κ-carrageenan, protein–polysaccharide complexes are formed, resulting in a gel-like sediment [22]. At the polysaccharide/protein ratio of 0.3, all protein and polysaccharide molecules are bonded into these complexes, leading to the absence or only minor traces of both species in the supernatant. At this ratio, the charge compensation between the native HEWL and κ-carrageenan is achieved. Upon the further addition of polysaccharide, the composition of the complexes does not change, indicating that complexes are stoichiometric [19]. The excess of polysaccharide remains in solution, and the spectra of sediments remain identical, providing evidence of the similarity of biopolymer structures in complexes (Figure 2A). Importantly, the formation of stoichiometric complexes is accompanied by the protein structure’s conversion from fibrillary to native-like. The component resulting from the amyloid cross-β structure at 1621 cm^−1^ disappears with subsequent raise of absorbance from the intramolecular β-structure at 1640 cm^−1^, with retention of the α-helices’ absorbance at 1655 cm^−1^ (Figure 2B). The shape of the resultant spectra of the complexes upon the mixing of HEWL fibrils and κ-carrageenan matches those of the complexes between native HEWL and κ-carrageenan (Figure 2C). The AFM images confirmed the absence of fibrillary structures in the resultant stoichiometric complexes at the meso-scale level [22]. From this observation, we can conclude that HEWL fibrils disaggregate under the formation of stoichiometric complexes with κ-carrageenan with the subsequent renaturation of proteins.

In contrast, the mixing of HEWL fibrils and κ-carrageenan below the stoichiometric polysaccharide-to-protein ratio (e.g., 0.1 at Figure 2A) results in formation of non-stoichiometric complexes. Comparing the integral intensity of polysaccharide absorbance between 1100 and 1000 cm^−1^ with that of the protein Amide I band allows the estimation of their relative content in complexes, which amounts to 20% protein excess at the 0.1 ratio over that at the 0.3 ratio (Table S1). Despite the complexes’ formation, they are not stoichiometric, which may be due to large fibril size. Accordingly, the spectra of HEWL in non-stoichiometric complexes show the preservation of some cross-β structures (Figure 2B and Figure S2 and Table S2). 

This probably indicates that the polysaccharide interacts with the fibril surface rather than with individual protein molecules and implies that the stability of fibrils stems from collective interactions. Nevertheless, some perturbations of protein secondary structures could be noted in non-stoichiometric complexes (see Figure S1 and Table S2 for spectra decomposition). The band of cross-β sheets decreases by 13% and shifts down to 1621 cm^−1^, which suggests more tight packing in fibrils [56]. At the same time, the absorbance of intramolecular β-structures at 1640 cm^−1^ increases by 23%, and helical content decreases by 8%.

The appearance of intramolecular β-structures in complexes at a ratio of 0.1 is in line with the observation that κ-carrageenan has higher affinity to β-structures than to the α-motive of HEWL [19]. Since the content of intramolecular β-structures increases from the non-stoichiometric complexes to stoichiometric ones from 33% to 45%, we suggest that it may act as an intermediate state in the course of conversion between the cross-β aggregative structures and native-like structures. 

Summing up, the efficiency of κ-carrageenan in the conversion of the protein cross-β structures to the native-like motives is regulated by the charge compensation of protein and polysaccharide complexes, which is complete at the stoichiometric ratio.

### 2.3. HEWL Fibril Mixtures with Flexible ι- or λ-Carrageenans

**Figure 3:**
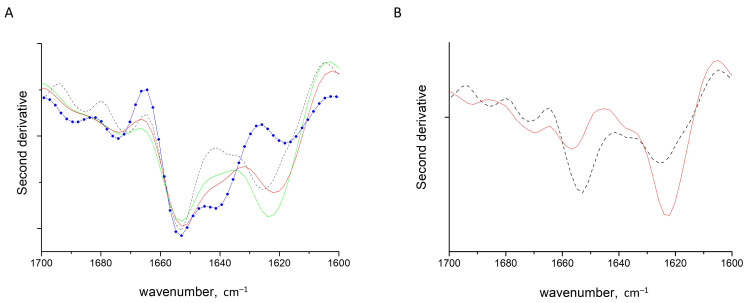
Second derivative spectra of HEWL fibrils (dashed line) and of their gel-like complexes with carrageenans at a polysaccharide-to-protein ratio of 0.3: (**A**) coiled κ- (blue line with symbols), ι- (red line) and λ- (green line) carrageenans, (**B**) κ-carrageenan in helical conformation (red line).

To reveal the charge effects on the HEWL fibrils’ conversion upon interaction with polysaccharides, we studied two other members of carrageenan family, ι- and λ-carrageenan, both in the coiled form. All three polysaccharides used in this study have different charge densities spread on different sulfation positions: κ-carrageenan has one sulfate group per disaccharide unit at galactosyl O-4, ι-carrageenan has two sulfate groups at galactosyl O-4 and at anhydrogalactosyl O-2 and λ-carrageenan has three sulfate groups per disaccharide, one at galactosyl O-6 and two at galactosyl O-2 (Figure 1). Importantly, two extreme cases of sulfation, κ-carrageenan and λ-carrageenan, form stoichiometric complexes with HEWL at a ratio of 0.3 [57,58]. All three polysaccharides show the ability to perturb fibril structures, but in different fashions. As was described in the previous section, κ-carrageenan increases the content of native secondary structures at the expense of cross-β structures. In contrast, λ-carrageenan induces a slight increase in the intermolecular parallel β-structures at 1623 cm^–1^, along with a raise of intramolecular β-sheets at 1640 cm^–1^ at the expense of α-helices (Figure S1). ι-Carrageenan has only minor effect on the shape of the Amide I band (Figure 3A). The detailed analysis reveals the conversion of some cross-β structures to intramolecular ones, amounting to no more than ~5% (Figure S1). The redshift of the cross-β structure band, together with the intensity raise of the intramolecular β-structures of HEWL in complexes with both ι-carrageenan and λ-carrageenan, resembles those seen upon the formation of non-stoichiometric complexes with κ-carrageenan. This newly formed intramolecular β-structure may be considered as a transient conformation in the course of polypeptide chain re-folding.

Summing up, among the three polysaccharides studied, the least-sulfated κ-carrageenan, upon complexation with HEWL fibrils, shifts the balance between cross-β structures and native-like motives toward the native-like ones; the conformational transition develops through the formation of intermediate intramolecular β-structures. The most-sulfated λ-carrageenan shifts balance to the more extensive intermolecular β-structures. This observation is in line with the correlation found earlier for GAGs; namely, that highly sulfated GAGs induce the stabilization of cross-β structures and fibril formation [33]. Furthermore, it was shown for GAGs that the presence of the 6-O sulfate group is critical for the induction of amyloid β-structures, which is also a factor appearing in the use of λ-carrageenan.

### 2.4. HEWL Fibril–Rigid κ-Carrageenan Mixtures

To answer the question of to what extent the density and position of sulfation influence the conformational balance between the cross-β and native β-structures, we studied the effect of κ-carrageenan chains in the helical form on the model HEWL fibrils. The formation of κ-carrageenan helices was confirmed by FTIR spectroscopy for the polysaccharide alone and the polysaccharide mixed with HEWL fibrils in solution based on previously achieved assignments for coil and helical conformations of κ-carrageenan (Figure S2A) [54]. 

The spectrum of the complexes of HEWL fibrils and κ-carrageenan helices shows a drastic increase in the cross-β structure absorbance with a concomitant decrease in that of α-helices (Figure 3B). The effect of the κ-carrageenan helix on the HEWL cross-β structure stabilization is more prominent than the influence of highly sulfated λ-carrageenan, which may come from the appropriate grouping of sulfate moieties in the double helix [37]. This observation confirms that the dense sulfating pattern has a decisive effect on the balance shift towards cross-β structures, which was deduced from the previous section. Furthermore, comparing the effects of λ-carrageenan and κ-carrageenan double helix we can conclude that O-6 sulfation is not the main dominant factor of the cross-β structure stabilization, in contrary to what has been deduced for sulfated GAGs.

Furthermore, concerning the opposite effect of the coil and helical forms of κ-carrageenan on HEWL structure, we can also state the importance of κ-carrageenan conformation for its ability to shift the balance toward fibril’s disruption. The power of κ-carrageenan flexible chains to destabilize cross-β structures toward protein native-like conformations may have its origin in the tight interaction of the hydrophobic residues of HEWL with the hydrophobic anhydrogalactosyl groups of κ-carrageenan. The ability of the flexible polysaccharide chain to adjust its conformation to maximize contacts with the protein promotes the reorganization of the protein structure. Since they are organized as double helices, the hydrophobic anhydrogalactosyl residues of κ-carrageenan are hidden in the core of the polysaccharide and sulfate groups are exposed to the solvent. Furthermore, the helical elements of the polysaccharide are rigid; therefore, only point interactions with protein fibrils are possible. 

**Figure 4:**
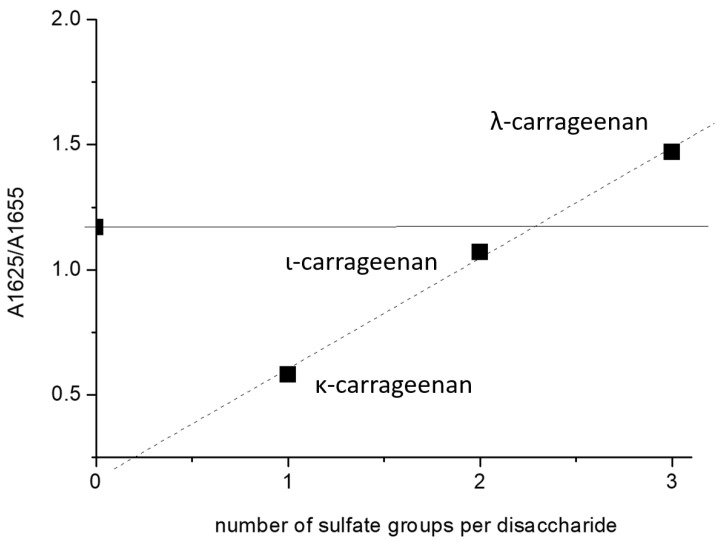
Correlation between the ratio of integral absorptions at 1625 and 1655 cm^−1^ of components of the Amide I band of HEWL in complex with carrageenans, differing in number of sulfate groups per disaccharide: κ- (1), ι- (2) and λ- (3). The ratio for pure amyloid fibrils is given at X = 0 and is marked a by horizontal line.

Figure 4 represents the ratio of the protein cross-β and helical structure absorbances in the presence of flexible carrageenan chains and shows that, in this case, the density of the polysaccharides’ sulfation is an adverse factor shifting the balance of protein conformation in favor of intermolecular amyloid cross-β. The double helix formation of κ-carrageenan increases the charge density twice. Nevertheless, the ratio of protein cross-β to helical absorbance (~2.8) outlies this dependency, suggesting that not only the charge density, but also the chain flexibility, is an important and outstanding factor.

### 2.5. HEWL Fibril–Chondroitin-4-Sulfate Mixtures

**Figure 5:**
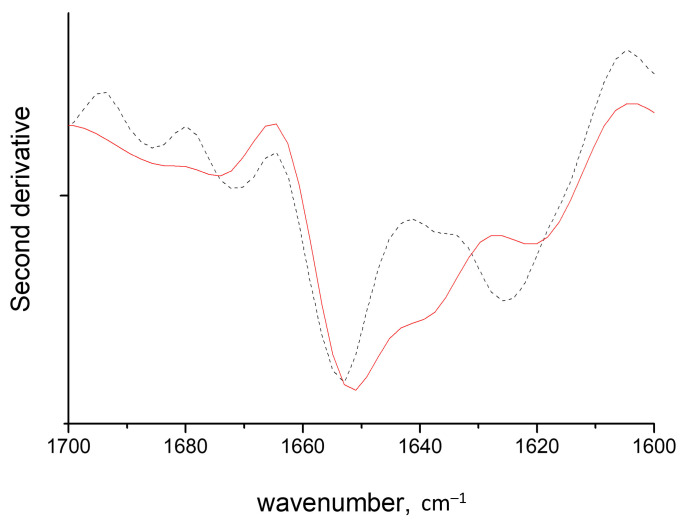
Second derivative spectra of HEWL fibrils (dashed line) and of their gel-like complexes with chondroitin-4-sulfate (red line) at a polysaccharide-to-protein ratio of 0.3.

To further unveil the role of sulfation at the disaccharide O-4 position and the presence of hydrophobic anhydrogalactosyl residue in the flexible polysaccharide chain in shifting the conformational balance from cross-β to native-like structures, we analyzed the effect of chondroitin-4-sulfate on the HEWL fibrils. The FTIR spectra demonstrate the conversion of cross-β structures to the native-like HEWL motives, resulted in an Amide I spectral shape similar to that described for stoichiometric complexes of HEWL with flexible κ-carrageenan (Figure 5). Despite the fact that chondroitin-4-sulfate is more charged than κ-carrageenan (Figure 1), the presence of the extra carboxylate group does not worsen the formation of native-like HEWL motives. The ratio of the 1625/1655 band areas in the spectrum of HEWL complexed with chondroitin-4-sulfate is of the same value as the ratio found in complexes with κ-carrageenan (Figure 4). Therefore, despite the absence of hydrophobic anhydrogalactosyl groups in chondroitin-4-sulfate, chain flexibility appears as a critical factor in its capability to define the balance between cross-β structures and native-like motives.

## 3. Materials and Methods

### 3.1. Materials and Fibril Preparation

Lyophilized HEWL powder was purchased from Sigma-Aldrich and used without preliminary purification. All polysaccharides were of commercial grade (κ-carrageenan (Sigma-Aldrich, Burlington, MA, USA, 22048, lot BCBT9953,), ι-carrageenan (Alfa Aesar, Ward Hill, MA, USA, J60603, lot T16F031), λ-re carrageenan (TCI Europe N.V., Zwijndrecht, Belgium, C3313, lot 46QNC-OT), and chondroitin-4-sulfate (Sigma-Aldrich C9819, lot SLCB0351). The molecular weights (Mp), determined by size exclusion chromatography, were 63 kDa for κ-carrageenan and 55 kDa for ι-carrageenan. Two monomolecular fractions of 55 kDa and 29 kDa were revealed for λ-carrageenan. 

The HEWL fibril preparation was as described in ref. [22]. Briefly, HEWL fibrils were prepared by incubating 5 mL of 15 mg∙mL^−1^ protein solution containing 250 mM NaCl at pH = 2 and 65 °C in a thermostat with stirring for 7 days. The fibrils’ growth kinetics were followed by thioflavin binding. The preparation of the water-soluble fraction was as follows: 1. fibrils were dialyzed against pure water until the convergence of conductivity changed in the dialysate; 2. samples were centrifuged; 3. sediment was discarded. The pH of the dialyzed lysozyme fibrils was 5.9. The morphology of the final fibrils at the meso-scale level was controlled using AFM images, as reported in ref. [22].

The concentration of the protein fibrils in the water-soluble fraction was estimated using a UV spectrophotometer at 280 nm and an extinction coefficient of ε_280_ = 2.65 mg∙mL^−1^∙cm^−1^ [59].

### 3.2. HEWL Fibril–Polysaccharide Mixing

First, aqueous solutions of polysaccharides were allowed to swell for one hour at 20 °C and then were stirred for two hours at 70 °C. The solutions were mixed with the supernatant fractions of HEWL fibrils with various mass polysaccharide-to-protein ratios. The final concentrations were 5 mg/mL for protein, and the pH of the mixtures was kept as ~5.9. To have κ-carrageenan in coiled conformation, the concentration of the stock solution was 5.5 mg/mL. To have it in helical conformation, a concentrated polysaccharide solution was used with a concentration of 11 mg/mL (with a concomitant increase in stock protein concentration).

### 3.3. FTIR Spectroscopy

The FTIR spectra were recorded using an IRAffinity-I spectrometer (Shimadzu, Europa GmbH, Duisburg, Germany) equipped with the attenuated total reflection (ATR) accessory and a ZnSe crystal. The spectra were recorded at 4 cm^−1^ resolution within the range of 4000–800 cm^−1^, and 256 scans were accumulated for each spectrum. 

The samples were placed directly on the surface of the ATR sensing element with a controlled constant temperature of 25 °C. To analyze the protein fine structure following from the Amide I band decomposition, the absorption of water in the vapor and liquid states was subtracted from the original spectra.

## 4. Conclusions

Using FTIR spectroscopy as a probe for structural order in both protein and polysaccharide, we have shown that the conformational balance between amyloid motives and native protein structures can be shifted via interactions with sulfated polysaccharides. This balance depends on both the density of sulfation and the flexibility of the polysaccharide chain, which act in an opposite fashion. In a sense of the recovery of native protein structures, flexible carrageenans form the following series: κ- > ι- > λ-. Less-charged κ-carrageenan, as well as chondroitin-4-sulfate, effectively converted HEWL amyloid secondary structures into native protein motives. More-sulfated ι-carrageenan is less effective in this manner, while the most charged polysaccharide, λ-carrageenan, even provokes an increase in amyloid motives. Chain flexibility seems to be a prerequisite of the ability of sulfated polysaccharides to recover native protein structures, as it stems from the opposite effect of rigid double helical κ-carrageenan, which drastically increased the amyloid motives.

## Data Availability

The data in this study are available on reasonable request from the corresponding author.

## Supplementary Materials

The supporting information can be downloaded at: https://www.mdpi.com/article/10.3390/ijms242216223/s1. Reference [60] is cited in the supplementary materials.

## References

[1] Fink A.L. (1998). Protein Aggregation: Folding Aggregates, Inclusion Bodies and Amyloid. Fold. Des..

[2] Sipe J.D., Benson M.D., Buxbaum J.N., Ikeda S.I., Merlini G., Saraiva M.J., Westermark P. (2016). Amyloid Fibril Proteins and Amyloidosis: Chemical Identification and Clinical Classification International Society of Amyloidosis 2016 Nomenclature Guidelines. Amyloid.

[3] Uversky V.N., Fink A.L. (2004). Conformational Constraints for Amyloid Fibrillation: The Importance of Being Unfolded. Biochim. Biophys. Acta.

[4] Brudar S., Hribar-Lee B. (2021). Effect of Buffer on Protein Stability in Aqueous Solutions: A Simple Protein Aggregation Model. J. Phys. Chem. B.

[5] Pedersen J.T., Heegaard N.H. (2013). Analysis of Protein Aggregation in Neurodegenerative Disease. Anal. Chem..

[6] Ghahghaei A., Faridi N. (2009). Review: Structure of Amyloid Fibril in Diseases. J. Biomed. Sci. Eng..

[7] Ke P.C., Zhou R., Serpell L.C., Riek R., Knowles T.P.J., Lashuel H.A., Gazit E., Hamley I.W., Davis T.P., Fandrich M. (2020). Half a Century of Amyloids: Past, Present and Future. Chem. Soc. Rev..

[8] Giorgetti S., Greco C., Tortora P., Aprile F.A. (2018). Targeting Amyloid Aggregation: An Overview of Strategies and Mechanisms. Int. J. Mol. Sci..

[9] Holubová M., Štěpánek P., Hrubý M. (2021). Polymer Materials as Promoters/Inhibitors of Amyloid Fibril Formation. Colloid Polym. Sci..

[10] Alam P., Siddiqi K., Chturvedi S.K., Khan R.H. (2017). Protein Aggregation: From Background to Inhibition Strategies. Int. J. Biol. Macromol..

[11] Chiti F., Dobson C.M. (2017). Protein Misfolding, Amyloid Formation, and Human Disease: A Summary of Progress over the Last Decade. Annu. Rev. Biochem..

[12] Debnath K., Sarkar A.K., Jana N.R., Jana N.R. (2022). Inhibiting Protein Aggregation by Small Molecule-Based Colloidal Nanoparticles. Acc. Mater. Res..

[13] Albuquerque H.M.T., Nunes da Silva R., Pereira M., Maia A., Guieu S., Soares A.R., Santos C.M.M., Vieira S.I., Silva A.M.S. (2022). Steroid-Quinoline Hybrids for Disruption and Reversion of Protein Aggregation Processes. ACS Med. Chem. Lett..

[14] Johnson L., Faidra Angelerou M.G., Surikutchi B.T., Allen S., Zelzer M., Marlow M. (2019). Low Molecular Weight Nucleoside Gelators: A Platform for Protein Aggregation Inhibition. Mol. Pharm..

[15] Peydayesh M., Kistler S., Zhou J., Lutz-Bueno V., Victorelli F.D., Meneguin A.B., Spósito L., Bauab T.M., Chorilli M., Mezzenga R. (2023). Amyloid-Polysaccharide Interfacial Coacervates as Therapeutic Materials. Nat. Commun..

[16] Makshakova O.N., Zuev Y.F. (2022). Interaction-Induced Structural Transformations in Polysaccharide and Protein-Polysaccharide Gels as Functional Basis for Novel Soft-Matter: A Case of Carrageenans. Gels.

[17] Derkach S.R., Voron’ko N.G., Kuchina Y.A., Kolotova D.S., Gordeeva A.M., Faizullin D.A., Gusev Y.A., Zuev Y.F., Makshakova O.N. (2018). Molecular Structure and Properties of Kappa-Carrageenan-Gelatin Gels. Carbohydr. Polym..

[18] Makshakova O., Antonova M., Bogdanova L., Faizullin D., Zuev Y. (2023). Regulation of Intersubunit Interactions in Homotetramer of Glyceraldehyde-3-Phosphate Dehydrogenases Upon Its Immobilization in Protein-Kappa-Carrageenan Gels. Polymers.

[19] Makshakova O.N., Bogdanova L.R., Faizullin D.A., Ermakova E.A., Zuev Y.F., Sedov I.A. (2021). Interaction-Induced Structural Transformation of Lysozyme and Kappa-Carrageenan in Binary Complexes. Carbohydr. Polym..

[20] Wang X., Nian Y., Zhang Z., Chen Q., Zeng X., Hu B. (2019). High Internal Phase Emulsions Stabilized with Amyloid Fibrils and Their Polysaccharide Complexes for Encapsulation and Protection of Β-Carotene. Colloids Surf. B Biointerfaces.

[21] Knowles T.P.J., Mezzenga R. (2016). Amyloid Fibrils as Building Blocks for Natural and Artificial Functional Materials. Adv. Mater..

[22] Makshakova O., Bogdanova L., Faizullin D., Khaibrakhmanova D., Ziganshina S., Ermakova E., Zuev Y., Sedov I. (2023). The Ability of Some Polysaccharides to Disaggregate Lysozyme Amyloid Fibrils and Renature the Protein. Pharmaceutics.

[23] Iannuzzi C., Irace G., Sirangelo I. (2015). The Effect of Glycosaminoglycans (Gags) on Amyloid Aggregation and Toxicity. Molecules.

[24] Liang Y., Ueno M., Zha S., Okimura T., Jiang Z., Yamaguchi K., Hatakeyama T., Oda T. (2021). Sulfated Polysaccharide Ascophyllan Prevents Amyloid Fibril Formation of Human Insulin and Inhibits Amyloid-Induced Hemolysis and Cytotoxicity in Pc12 Cells. Biosci. Biotechnol. Biochem..

[25] Shi D., Sheng A., Chi L. (2021). Glycosaminoglycan-Protein Interactions and Their Roles in Human Disease. Front. Mol. Biosci..

[26] Magnus J.H., Husby G., Kolset S.O. (1989). Presence of Glycosaminoglycans in Purified Aa Type Amyloid Fibrils Associated with Juvenile Rheumatoid Arthritis. Ann. Rheum. Dis..

[27] Torres-Bugeau C.M., Ávila C.L., Raisman-Vozari R., Papy-Garcia D., Itri R., Barbosa L.R., Cortez L.M., Sim V.L., Chehín R.N. (2012). Characterization of Heparin-Induced Glyceraldehyde-3-Phosphate Dehydrogenase Early Amyloid-Like Oligomers and Their Implication in A-Synuclein Aggregation. J. Biol. Chem..

[28] Perez S., Makshakova O., Angulo J., Bedini E., Bisio A., de Paz J.L., Fadda E., Guerrini M., Hricovini M., Hricovini M. (2023). Glycosaminoglycans: What Remains to Be Deciphered?. JACS Au.

[29] Bravo R., Arimon M., Valle-Delgado J.J., García R., Durany N., Castel S., Cruz M., Ventura S., Fernàndez-Busquets X. (2008). Sulfated Polysaccharides Promote the Assembly of Amyloid Beta(1-42) Peptide into Stable Fibrils of Reduced Cytotoxicity. J. Biol. Chem..

[30] Takase H., Tanaka M., Yamamoto A., Watanabe S., Takahashi S., Nadanaka S., Kitagawa H., Yamada T., Mukai T. (2016). Structural Requirements of Glycosaminoglycans for Facilitating Amyloid Fibril Formation of Human Serum Amyloid A. Amyloid.

[31] Cohlberg J.A., Li J., Uversky V.N., Fink A.L. (2002). Heparin and Other Glycosaminoglycans Stimulate the Formation of Amyloid Fibrils from Alpha-Synuclein in Vitro. Biochemistry.

[32] Mehra S., Ghosh D., Kumar R., Mondal M., Gadhe L.G., Das S., Anoop A., Jha N.N., Jacob R.S., Chatterjee D. (2018). Glycosaminoglycans Have Variable Effects on A-Synuclein Aggregation and Differentially Affect the Activities of the Resulting Amyloid Fibrils. J. Biol. Chem..

[33] Snow A.D., Cummings J.A., Lake T. (2021). The Unifying Hypothesis of Alzheimer’s Disease: Heparan Sulfate Proteoglycans/Glycosaminoglycans Are Key as First Hypothesized over 30 Years Ago. Front. Aging Neurosci..

[34] Choudhary S., Save S.N., Vavilala S.L. (2018). Unravelling the Inhibitory Activity of Chlamydomonas Reinhardtii Sulfated Polysaccharides against Alpha-Synuclein Fibrillation. Sci. Rep..

[35] Running C.A., Falshaw R., Janaswamy S. (2012). Trivalent Iron Induced Gelation in Lambda-Carrageenan. Carbohydr. Polym..

[36] Schefer L., Adamcik J., Mezzenga R. (2014). Unravelling Secondary Structure Changes on Individual Anionic Polysaccharide Chains by Atomic Force Microscopy. Angew. Chem. Int. Ed..

[37] Anderson N.S., Campbell J.W., Harding M.M., Rees D.A., Samuel J.W.B. (1969). X-Ray Diffraction Studies of Polysaccharide Sulphates: Double Helix Models for Κ- and Ι-Carrageenans. J. Mol. Biol..

[38] Ikeda S., Morris V.J., Nishinari K. (2001). Microstructure of Aggregated and Nonaggregated Kappa-Carrageenan Helices Visualized by Atomic Force Microscopy. Biomacromolecules.

[39] Swaminathan R., Ravi V.K., Kumar S., Kumar M.V., Chandra N. (2011). Lysozyme: A Model Protein for Amyloid Research. Adv. Protein Chem. Struct. Biol..

[40] Eanes E.D., Glenner G.G. (1968). X-ray Diffraction Studies on Amyloid Filaments. J. Histochem. Cytochem..

[41] Willbold D., Strodel B., Schroder G.F., Hoyer W., Heise H. (2021). Amyloid-Type Protein Aggregation and Prion-Like Properties of Amyloids. Chem. Rev..

[42] Islam Z., Ali M.H., Popelka A., Mall R., Ullah E., Ponraj J., Kolatkar P.R. (2021). Probing the Fibrillation of Lysozyme by Nanoscale-Infrared Spectroscopy. J. Biomol. Struct. Dyn..

[43] Zurdo J., Guijarro J.I., Dobson C.M. (2001). Preparation and Characterization of Purified Amyloid Fibrils. J. Am. Chem. Soc..

[44] Ruggeri F.S., Longo G., Faggiano S., Lipiec E., Pastore A., Dietler G. (2015). Infrared Nanospectroscopy Characterization of Oligomeric and Fibrillar Aggregates During Amyloid Formation. Nat. Commun..

[45] Sarroukh R., Goormaghtigh E., Ruysschaert J.M., Raussens V. (2013). Atr-Ftir: A “Rejuvenated” Tool to Investigate Amyloid Proteins. Biochim. Biophys. Acta.

[46] Ermakova E.A., Makshakova O.N., Zuev Y.F., Sedov I.A. (2021). Fibril Fragments from the Amyloid Core of Lysozyme: An Accelerated Molecular Dynamics Study. J. Mol. Graph. Model..

[47] Wei S., Li Y., Li K., Zhong C. (2022). Biofilm-Inspired Amyloid-Polysaccharide Composite Materials. Appl. Mater. Today.

[48] Usuelli M., Germerdonk T., Cao Y., Peydayesh M., Bagnani M., Handschin S., Nystrom G., Mezzenga R. (2021). Polysaccharide-Reinforced Amyloid Fibril Hydrogels and Aerogels. Nanoscale.

[49] Dong A., Huang P., Caughey W.S. (1990). Protein Secondary Structures in Water from Second-Derivative Amide I Infrared Spectra. Biochemistry.

[50] Susi H., Byler D.M. (1983). Protein Structure by Fourier Transform Infrared Spectroscopy: Second Derivative Spectra. Biochem. Biophys. Res. Commun..

[51] Kačuráková M., Capek P., Sasinková V., Wellner N., Ebringerová A. (2000). Ft-Ir Study of Plant Cell Wall Model Compounds: Pectic Polysaccharides and Hemicelluloses. Carbohydr. Polym..

[52] McCann M.C., Hammouri M., Wilson R., Belton P., Roberts K. (1992). Fourier Transform Infrared Microspectroscopy Is a New Way to Look at Plant Cell Walls. Plant. Physiol..

[53] Sekkal M., Legrand P., Huvenne J.P., Verdus M.C. (1993). The Use of Ftir Microspectrometry as a New Tool for the Identification in Situ of Polygalactanes in Red Seaweeds. J. Mol. Struct..

[54] Makshakova O.N., Faizullin D.A., Zuev Y.F. (2020). Interplay between Secondary Structure and Ion Binding Upon Thermoreversible Gelation of Kappa-Carrageenan. Carbohydr. Polym..

[55] Belton P.S., Goodfellow B.J., Wilson R.H. (1989). A Variable-Temperature Fourier-Transform Infrared Study of Gelation in Ι- and Κ-Carrageenans. Macromolecules.

[56] Roeters S.J., Iyer A., Pletikapić G., Kogan V., Subramaniam V., Woutersen S. (2017). Evidence for Intramolecular Antiparallel Beta-Sheet Structure in Alpha-Synuclein Fibrils from a Combination of Two-Dimensional Infrared Spectroscopy and Atomic Force Microscopy. Sci. Rep..

[57] Antonov Y.A., Zhuravleva I.L. (2019). Complexation of Lysozyme with Lambda Carrageenan: Complex Characterization and Protein Stability. Food Hydrocoll..

[58] Antonov Y.A., Zhuravleva I.L., Cardinaels R., Moldenaers P. (2018). Macromolecular Complexes of Lysozyme with Kappa Carrageenan. Food Hydrocoll..

[59] Pace C.N., Vajdos F., Fee L., Grimsley G., Gray T. (1995). How to Measure and Predict the Molar Absorption Coefficient of a Protein. Protein Sci..

[60] Barth A. (2007). Infrared Spectroscopy of Proteins. Biochim. Biophys. Acta (BBA) -Bioenerg..

