# Supplementary material for: Sulfated Polysaccharides as a Fighter with Protein Non-Physiological Aggregation: The Role of Polysaccharide Flexibility and Charge Density
Source: Int J Mol Sci. 2023 Nov 12;24(22):16223. doi: 10.3390/ijms242216223 (PMC10671430; doi:10.3390/ijms242216223)
Supplement: Supplementary file 1 [file ijms-24-16223-s001.zip › ijms-2654729-supplementary.pdf]

Supplemental Material

Sulfated Polysaccharides as a Fighter with Protein Non-Physiological Aggregation: The Role of Polysaccharide Flexibility and Charge Density

Olga N. Makshakova *, Liliya R. Bogdanova, Dzhigangir A. Faizullin, Elena A. Ermakova and Yuriy F. Zuev

Kazan Institute of Biochemistry and Biophysics, FRC Kazan Scientific Center of RAS, 2/31 Lobachevsky Str., 420111, Kazan, Russia
* Correspondence: olga.makshakova@kibb.knc.ru

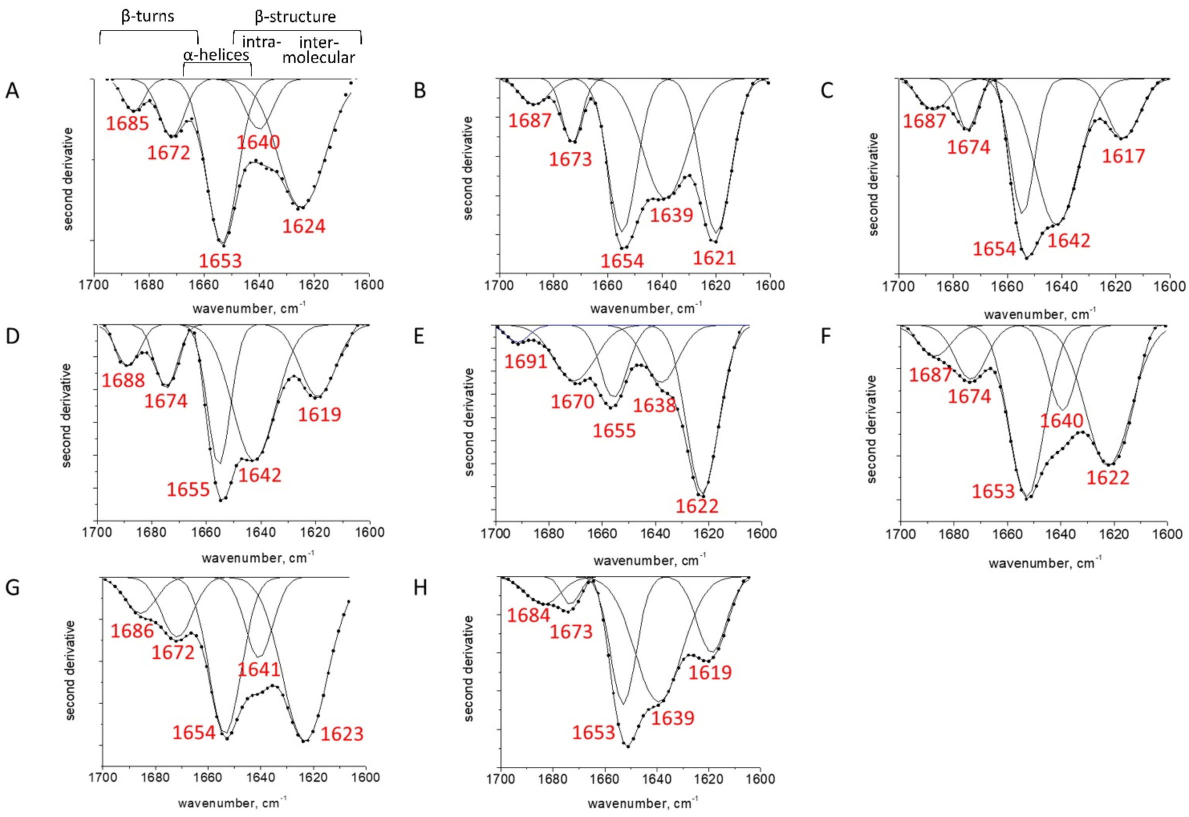

Figure S1. Amide I fitting with individual Gaussian components in second-derivative spectra of initial HEWL fibrils solution (A), gel-like complexes of HEWL fibrils and coiled κ-carrageenans with different initial polysaccharide-to-protein ratios: 0.1 (B), 0.3 (C), 0.6 (D), gel-like complexes of HEWL fibrils and ι-carrageenan (E), λ-carrageenan (F), helical κ-carrageenan (G), chondroitin-4-sulfate (H). .

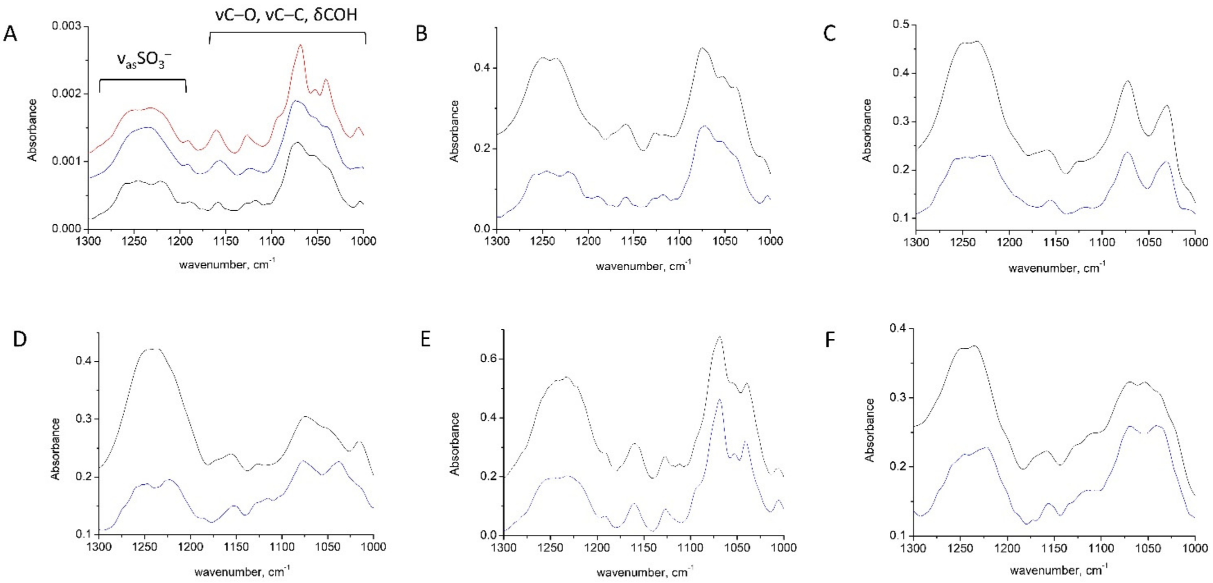

Figure S2. Absorbance spectra of 0.5% solution of κ-carrageenan in coil conformation (black), 2% sol of temperature-denaturated κ-carrageenan in coil conformation (blue), and 2% gel of κ-carrageenan in helical conformation (red) (A), gel-like complexes of HEWL fibrils with polysaccharides (black) and related spectra of pure polysaccharides (blue) for κ-carrageenan in coil conformation (B), ι-carrageenan (C), λ-carrageenan (D), κ-carrageenan in helical conformation (E) and chondroitin-4-sulfate (F).

Table S1. Band areas for protein absorption and polysaccharide absorption in complexes of HEWL fibrils and κ-carrageenan (see spectra in Figure 2 A of main text).

Polysaccharide-to-protein ratio	S1 (1700–1600cm^{-1})	S2 (1100–1000cm^{-1})	S1/S2
0.1	54	13	4.2
0.3	52	15	3.5
0.6	52	15	3.5

\

Table S2. Some characteristics of Amide I fitting with individual Gaussian components in second-derivative spectra of molecular systems studied (see spectra in Figure S1).

Sample	Wavenumber, cm^{-1}	Halfwidth, cm^{-1}	Peak area, %	Peak attribution
Fibrils	1624	20	40	
κ-carrageenan				
0.1	1621	13	28	
0.3	1617	14	14	
0.6	1619	14	17	
ι-carrageenan	1622	20	37	amyloid β-structure
λ-carrageenan	1623	19	42	+ side chains*
κ-carrageenan (helical)	1622	14	46	
Chondroitin-4-sulfate	1619	13	17	
Fibrils	1653	13	35	
κ-carrageenan				
0.1	1654	12	27	
0.3	1654	10	24	
0.6	1655	10	23	
ι-carrageenan	1653	15	34	
λ-carrageenan	1654	14	29	α-helix
κ-carrageenan (helical)	1655	12	16	
Chondroitin-4-sulfate	1653	11	25	
Fibrils	1640	11	9	
κ-carrageenan				
0.1	1639	19	34	
0.3	1642	18	47	
0.6	1642	18	42	
ι-carrageenan	1640	12	13	intramolecular
λ-carrageenan	1641	12	13	β-structure
κ-carrageenan (helical)	1638	15	16	
Chondroitin-4-sulfate	1639	21	46	

*side chain absorbance usually appears as a minor band and extends from 1609 to 1620 cm^{-1} [60]

[60] Barth, A. Infrared Spectroscopy of Proteins. *Biochimica et Biophysica Acta (BBA) - Bioenergetics* **2007**, *1767*, 1073-1101, doi:https://doi.org/10.1016/j.bbabio.2007.06.004.
